# Evaluation of the effect of intrathecal GM1 in 24, 48, and 72 hours after acute spinal cord injury in rats

**DOI:** 10.1016/j.clinsp.2023.100228

**Published:** 2023-07-05

**Authors:** Daniel de Moraes Ferreira Jorge, Raphael Martus Marcon, Alexandre Fogaça Cristante, Tarcísio Eloy Pessoa Barros Filho, Gustavo Bispo dos Santos

**Affiliations:** Instituto de Ortopedia e Traumatologia, Hospital das Clinicas, Faculdade de Medicina, Universidade de São Paulo (IOT-HC/FMUSP), São Paulo, SP, Brazil

**Keywords:** Spinal cord injuries, G(M1) ganglioside, Injections, Spinal, Rats, Wistar

## Abstract

**Objective:**

The aim of this study was to evaluate the best timing and feasibility of intrathecal application of sodium monosialoganglioside (GM1) after spinal cord contusion in Wistar rats as an experimental model.

**Methods:**

Forty Wistar rats were submitted to contusion spinal cord injury after laminectomy. The animals were randomized and divided into four groups: Group 1 – Intrathecal application of GM1 24 hours after contusion; Group 2 – Intrathecal application of GM1 48 hours after contusion; Group 3 – intrathecal application of GM1 72 hours after contusion; Group 4 – Sham, with laminectomy and intrathecal application of 0.5 mL of 0.9% saline solution, without contusion. The recovery of locomotor function was evaluated at seven different moments by the Basso, Beattie, and Bresnahan (BBB) test. They were also assessed by the horizontal ladder, with sensory-motor behavioral assessment criteria, pre-and postoperatively.

**Results:**

This experimental study showed better functional scores in the group submitted to the application of GM1, with statistically significant results, showing a mean increase when evaluated on known motor tests like the horizontal ladder and BBB, at all times of evaluation (p < 0.05), especially in group 2 (48 hours after spinal cord injury). Also, fewer mistakes and slips over the horizontal ladder were observed, and many points were achieved at the BBB scale analysis.

**Conclusion:**

The study demonstrated that the intrathecal application of GM1 after spinal cord contusion in Wistar rats is feasible. The application 48 hours after the injury presented the best functional results.

## Highlights

• GM1 has neuroprotective effects, but it may face difficulty with the blood-brain barrier.
• Intrathecal application in models of spinal cord injury induced in rats.
• Determination of optimal timing of GM1 application for best functional results.

## Introduction

Spinal cord injury represents about 11,000 new cases annually in Brazil, mainly affecting young adults during their productive phase.^1^^,^^2^ Traumatic spinal cord injury causes damage that can be divided into two phases: initially, necrotic cell death occurs in the injured site due to mechanical stress,^3^ then a secondary injury is followed in the second phase, which reaches the adjacent tissue of the primary injury and causes apoptosis.^4^ In addition, secondary injury induces an inflammatory response, edema, reduced blood flow, and increased glutamate production in the spinal cord due to neurochemical changes that occur minutes to days after injury.^5^

The primary injury is irreversible; therefore, the reduction of the secondary injury is fundamental to promote axonal regeneration, and restrict the amplitude of damage, since with the increased production of glutamate, higher levels of sodium and calcium influx in neurons will occur, expanding the area of demyelination and cellular apoptosis.^6^

Currently, there is no pharmacological strategy that presents real benefit. Although high doses of methylprednisolone are still administered, the evidence proving its effectiveness is very tenuous, and the side effects of treatment are significant.^7^, ^8^, ^9^

Experimental studies in animals suggest using different therapeutic agents, such as sodium monosialoganglioside (GM1), one of the main glycosphingolipids of mammalian nervous tissue and a potentiator of neurotrophic effects on neural regeneration. Several properties are associated with their use that enhances the recovery of functional connections by increasing the plasticity mechanisms of injured spinal cord circuits, urging the reduction of neuron destruction after trauma, and GM1 is already a therapeutic option for central nervous system injuries.^10^

The intrathecal injection technique is widely used to administer some drugs that do not cross the blood-brain barrier.^11^ Following this concept, the administration of GM1 was associated with the intrathecal injection technique since the molecule has difficulties overcoming the blood-brain barrier.^12^^,^^13^

The search to accelerate and intensify the natural process of neural regeneration has been long. The application of GM1 at an ideal time after a traumatic spinal injury associated with intrathecal administration may represent progress in the quality and speed of nerve regeneration. The aim of this study is to evaluate the best timing and feasibility of intrathecal application of GM1 after spinal cord contusion in Wistar rats as an experimental model.

## Methods

The study was submitted to and approved by the institutional ethics committee in animal research (1394/2019) and strictly followed the ethical guidelines and standards established by the Guidelines for Reporting Animal Research (ARRIVE).^14^

The experimental study was conducted at the Laboratório de Estudos de Traumatismo Raquimedular e Nervos of the Instituto de Ortopedia e Traumatologia da Faculdade de Medicina da Universidade de São Paulo (LETRAN, IOT-FMUSP).

At the end of the study, the animals were euthanized, following the legislation and precepts of the Brazilian College of Animal Experimentation (COBEA) ^15^ and obeying the protocol published in “Euthanasia Practice Guideline of the National Council for the Control of Animal Experimentation (CONCEA)”.^16^

### Experimental groups

Forty male Wistar rats were selected, all from a single supplier, from the Centro de Bioterismo da Faculdade de Medicina de São Paulo, with a mean age of 12 weeks and weight between 250g and 450g, with normal clinical status and normal initial motor function, according to the Basso, Beattie, and Bresnahan (BBB) criteria.^17^^,^^18^ The rats were kept in individual cages, with control of temperature, humidity, air filtration, water supply, and *ad libitum*.

The animals were randomly divided sequentially into four groups of ten animals and submitted to controlled spinal cord contusion:

Group 1: 24 hours after contusion, an intrathecal dose of GM1 (30 mg/kg) was given;
Group 2: 48 hours after contusion, an intrathecal dose of GM1 (30 mg/kg) was given;
Group 3: 72 hours after contusion, an intrathecal dose of GM1 (30 mg/kg) was given;
Group 4: Sham, submitted to laminectomy and application of 0.5 mL of 0.9% saline solution, for control and standardization of the technique, without contusion.

Group 4 is used as a negative control of the surgical procedure and a negative control of the intervention when using 0.9% saline solution, avoiding an additional control group.

### Experimental model of spinal cord injury

To perform the spinal cord contusion, the animals underwent subcutaneous injection of tramadol hydrochloride and 5 mg/kg pentabiotic one hour before surgery. The anesthesia protocol was performed with isoflurane (1.5V%‒2.0V%) in 100% oxygen. After mild sedation, a mask was placed, covering the entire animal's face, leading to a deeper anesthetic condition.

**Fig. 1:**
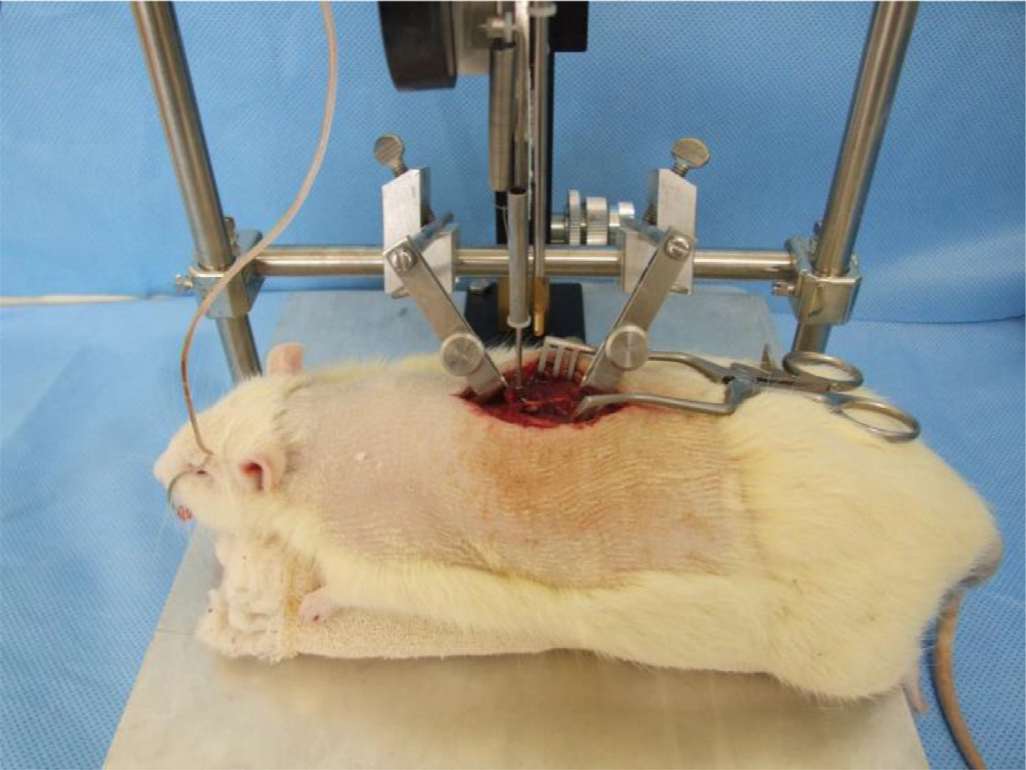
Animal positioned for spinal cord contusion and initial contact of the equipment with the spinal cord.

Spinal cord exposure for controlled contusion was performed with a surgical microscope (Fig. 1). After trichotomy, an incision was made dorsally midline to expose the posterior elements of the spine, from T8 to T12.

The muscles inserted in the spinous processes and the laminae from T9 to T11 were detached from their osseous insertions. Next, the spinous process and the laminae from T8 were removed with a bone rongeur until the spinal cord exposure and the positioning of the tip of the NYU-Impactor (New York University, 1993).^19^ After positioning the animal, the equipment was properly calibrated at a height of 12.5 mm, causing a moderate contusion.^18^

After confirmation of spinal cord contusion, the site was checked and washed with saline sodium chloride solution at room temperature. A paravertebral muscular en block suture was performed, then a cutaneous suture with mono-nylon 2.0.

### Standardization of the intrathecal application technique

**Fig. 2:**
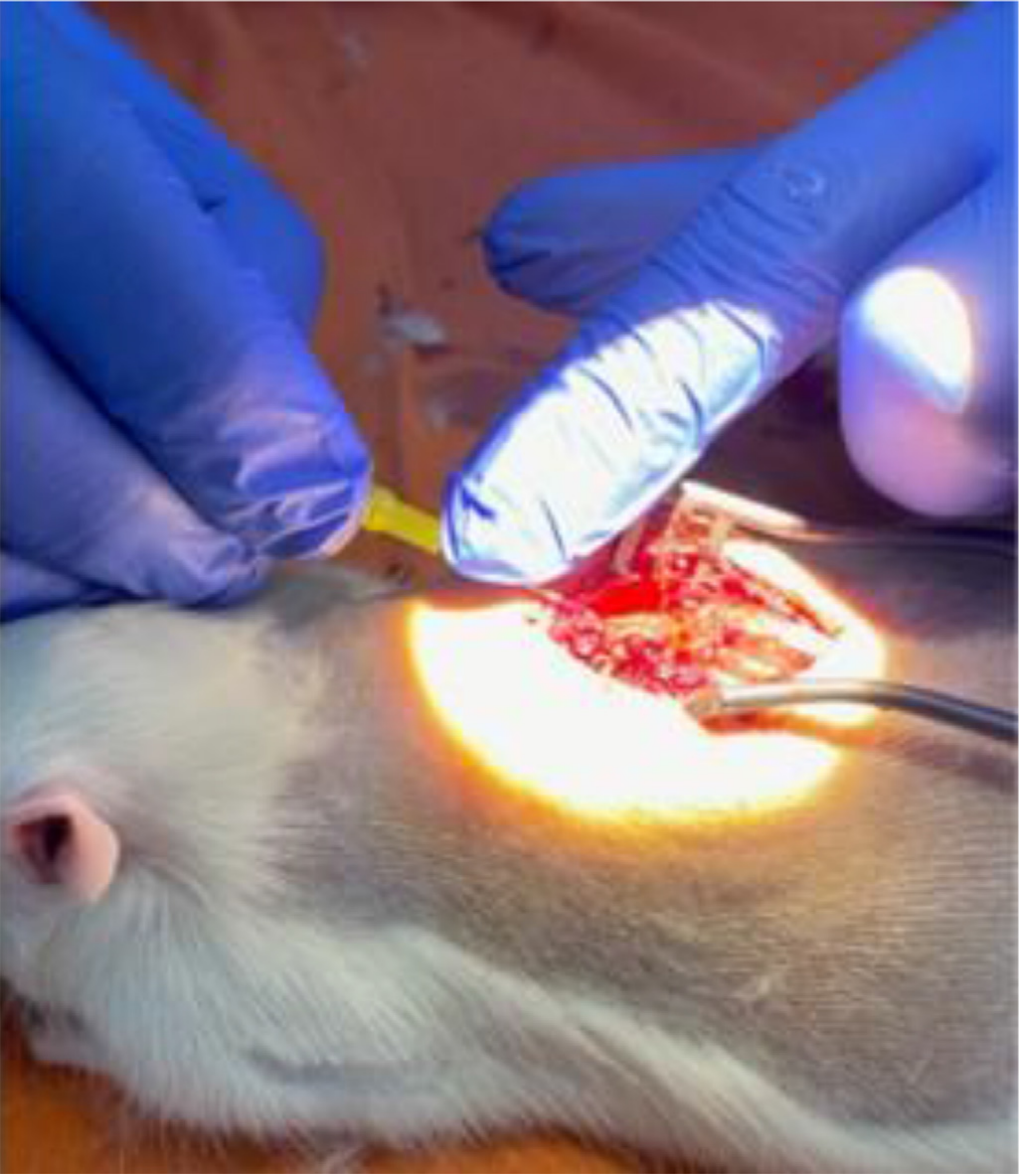
Intrathecal application under microscope vision.

An intrathecal application technique similar to spinal anesthesia was used to administer the drug directly to the central nervous system, but in this case, laminectomy and direct visual exposure of the dura mater. First, the needle (4 × 0.23 mm) was inserted into the cerebrospinal fluid space, by microscope-assisted application, at the T8 level. Then 30 mg/kg of GM1 (0.5 mL) or 0.9% saline solution was injected into the control group (Fig. 2).

Group 4 was used as a control group to standardize the experimental model. After the laminectomy, without associated spinal cord injury, an intrathecal injection of 0.5 mL of 0.9% saline solution was performed under direct vision.

### Postoperative protocol

Animals underwent intraperitoneal antibiotic prophylaxis with 5 mg/kg cefazolin sodium during the surgical intervention (immediately after the contusion) and daily for three days. For pain relief, the rats were administered 2 mg/kg meloxicam once daily for seven days and 5 mg/100g tramadol intramuscularly for five days.

Urine was extracted daily from the animals, observing the presence of blood. The degree of dehydration by skin turgor was evaluated to verify the need for antibiotic therapy.

All groups were submitted to the protocol for the same period indiscriminately. Animals were observed to identify exclusion criteria and complications, such as mutilations, infections, or other alterations.

### Exclusion criteria

The exclusion criteria were: death after spinal cord injury, skin changes, autophagy or mutilation, deep infection and refractory to antibiotic therapy after an injury, urine infection diagnosed by the presence of blood in the urine refractory to antibiotic treatment for ten days, normal mobility in the first evaluation after spinal cord injury in the groups undergoing experimental spinal cord injury (21 points on the functional BBB scale), loss > 10% of body weight after an injury.

### Functional assessment of locomotor capacity according to the BBB scale

The evaluation of the BBB scale^17^ always occurred at the same time and place, without identification between the groups (blind test) at seven different times, after two days of spinal cord injury, and weekly until the sixth postoperative week. The animal was then classified using a score ranging from 0 to 21, with 0 animals with total limb paraplegia and 21 with normal locomotor activity.^17^

The motor analyses were performed by the laboratory veterinarian Gustavo Bispo dos Santos, who is experienced in such evaluations and without information about which animals had been medicated.

### Horizontal ladder

A horizontal ladder 100 cm long, 35 cm wide, suspended 46 cm from the ground, and with a fixed space of 1.5 cm between each metal rung was used to evaluate the proprioceptive function of the animals.^20^ The animals were first trained to walk on the ladder for two days before surgery and had to cross it five times.

In the assessments, the animals had to walk voluntarily three times along the ladder. Then, the total number of steps, hits, slips, and errors was counted.

The hits consisted of correctly positioning the paws in the metal rung. The slips consisted of positioning the paw in the metal rung, followed by the fall of the paw between the rungs. Two errors were considered separately; dragging the hind paws along the horizontal ladder and positioning the paw between the metal rungs.^21^^,^^22^ The values of the three pass through the horizontal ladder were obtained for all types of answers (correct and errors).

### Statistical analysis

IBM-SPSS software for Windows version 22.0 (IBM Corp., Armonk, New York, USA) was used to perform the statistical analysis.

Descriptive statistics were performed following the experimental groups and the evaluation times using summary measures (mean, standard deviation, median, minimum and maximum) and compared the parameters using Generalized Evaluation Equations (EEG) with normal distribution and identity binding function, assuming AR^1^ correlation matrix between the evaluation times for the BBB scale and the horizontal ladder.^23^

Animal weight assessments were compared using analysis of variances (ANOVA), followed by multiple Bonferroni comparisons to identify between which groups and time points the differences occurred.^24^

## Results

The results are expressed in tables and graphs to facilitate and organize the analyses.

**Fig. 3:**
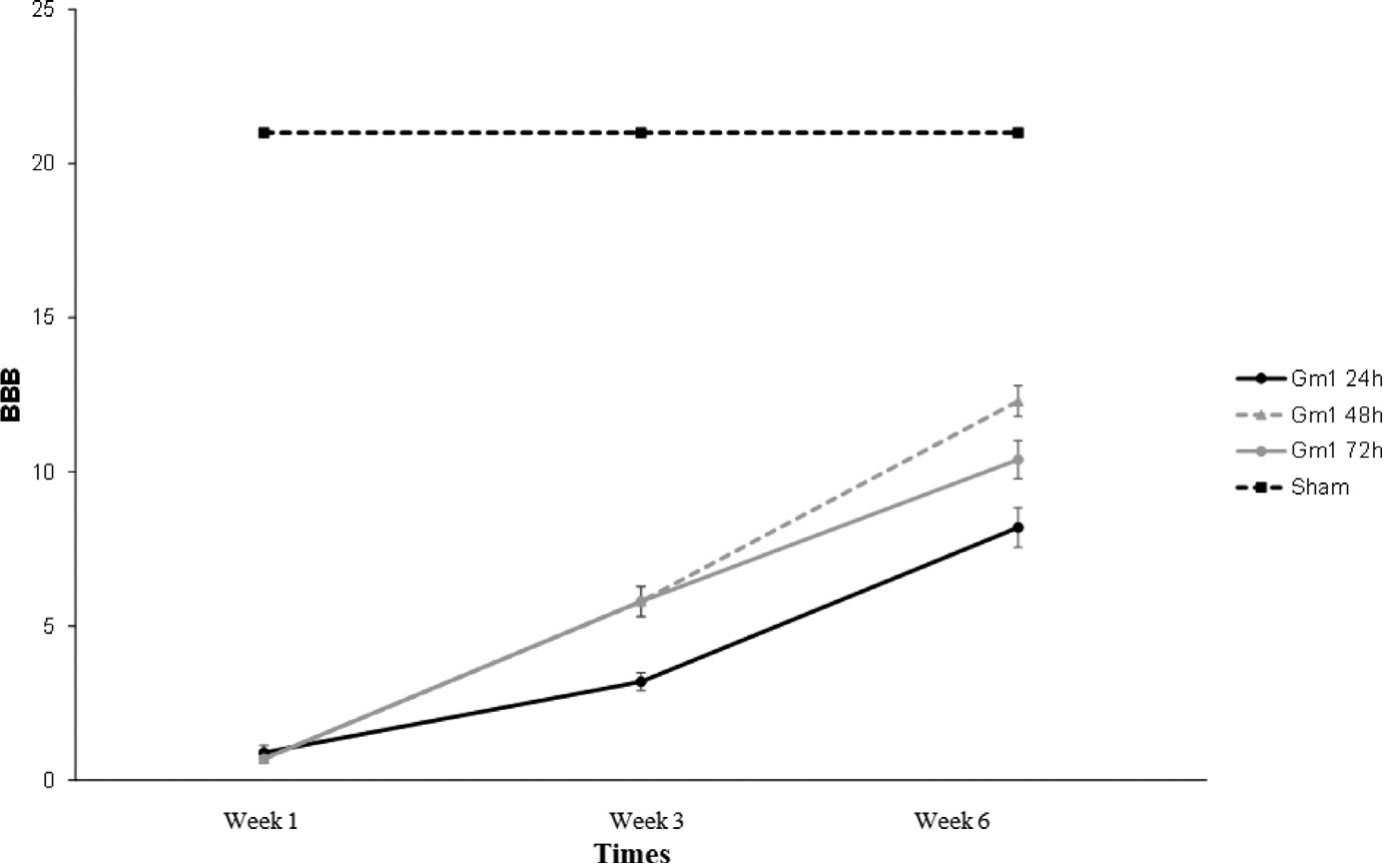
Mean values and respective standard errors of the BBB scale according to groups and times.

There was a statistical difference throughout the evaluation of the groups when evaluating the mean values of the BBB scale (p-interaction < 0.001). The results are shown in Fig. 3. Knowing that the BBB scale provides points for each functional capacity established, up to a maximum of 21, and that the higher points achieved, the better the quality of motor function of the animal is evaluated. Therefore, 0 is given to paraplegia and 21 to functionally normal animals.

The groups in which GM1 was administered showed a statistically significant mean increase when evaluated on the BBB scale at all times of evaluation (p < 0.05). In the first week, all groups were statistically equal on the BBB scale, and in the third and sixth weeks, the mean value in the GM1 48 hours and GM1 72 hours groups were statistically higher than in the GM1 24 hours group (p < 0.05). The results are shown in Table 1.

**Table 1:** Results of the multiple comparisons of BBB scale among the groups and times evaluated according to the differences found.

						(95%) IC	
Group/Time	Comparison		Mean difference	Standard error	p	Inferior	Superior
Gm1 24h	Week 1 - Week 3		-2.30	0.61	**0.006**	-4.26	-0.34
	Week 1 - Week 6		-7.30	0.62	**<0.001**	-9.27	-5.33
	Week 3 - Week 6		-5.00	0.61	**<0.001**	-6.96	-3.04
Gm1 48h	Week 1 - Week 3		-5.10	0.61	**<0.001**	-7.06	-3.14
	Week 1 - Week 6		-11.60	0.62	**<0.001**	-13.57	-9.63
	Week 3 - Week 6		-6.50	0.61	**<0.001**	-8.46	-4.54
Gm1 72h	Week 1 - Week 3		-5.10	0.61	**<0.001**	-7.06	-3.14
	Week 1 - Week 6		-9.70	0.62	**<0.001**	-11.67	-7.73
	Week 3 - Week 6		-4.60	0.61	**<0.001**	-6.56	-2.64
Week 1	Gm1 24h - Gm1 48h		0.20	0.62	>0.999	-1.77	2.17
	Gm1 24h - Gm1 72h		0.20	0.62	>0.999	-1.77	2.17
	Gm1 48h - Gm1 72h		0.00	0.62	>0.999	-1.97	1.97
Week 3	Gm1 24h - Gm1 48h		-2.60	0.62	**0.001**	-4.57	-0.63
	Gm1 24h - Gm1 72h		-2.60	0.62	**0.001**	-4.57	-0.63
	Gm1 48h - Gm1 72h		0.00	0.62	>0.999	-1.97	1.97
Week 6	Gm1 24h - Gm1 48h		-4.10	0.62	**<0.001**	-6.07	-2.13
	Gm1 24h - Gm1 72h		-2.20	0.62	**0.013**	-4.17	-0.23
	Gm1 48h - Gm1 72h		1.90	0.62	0.074	-0.07	3.87

Multiple comparisons of Bonferroni.

**Fig. 4:**
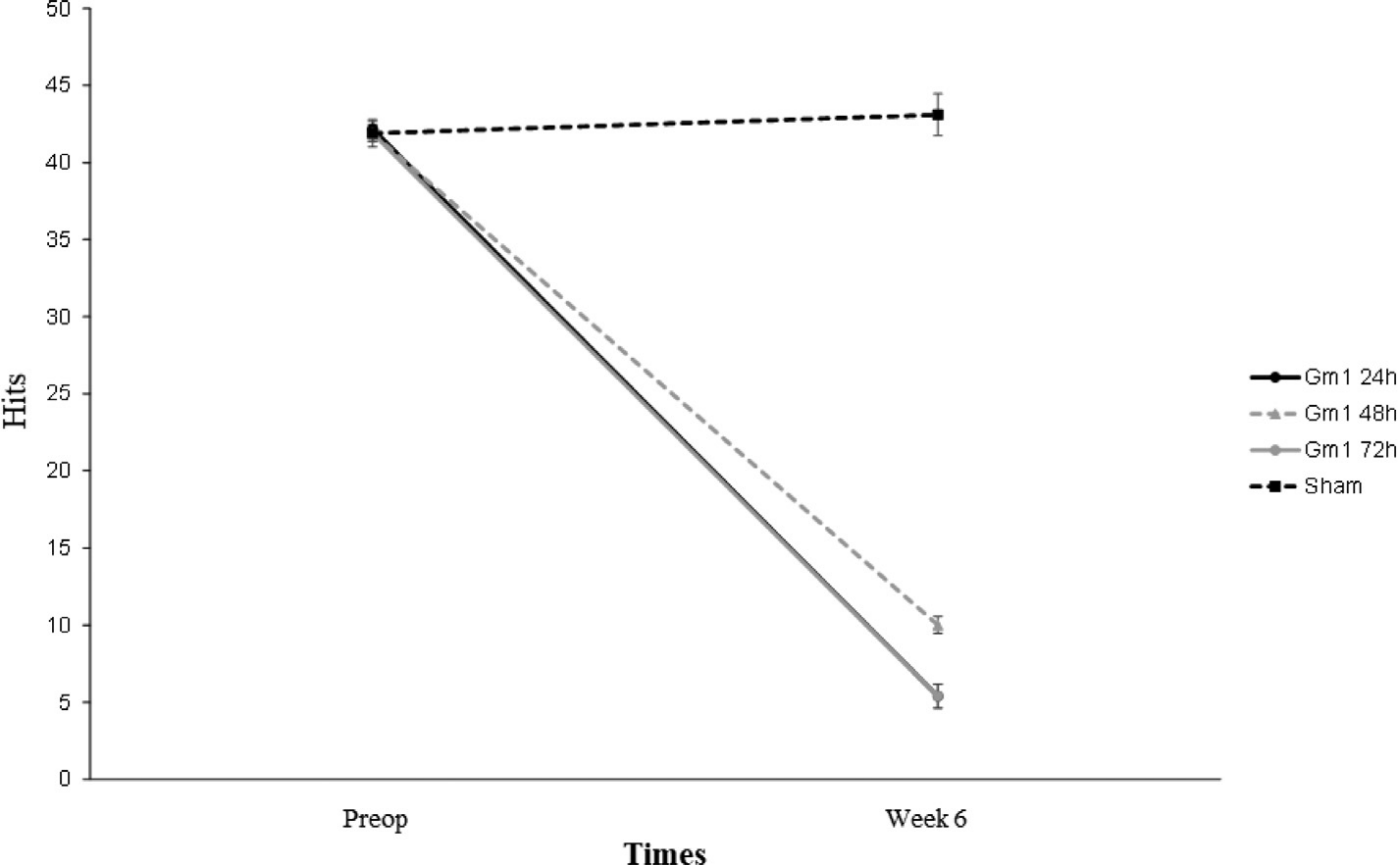
Mean values and respective standard errors of the hits on the horizontal ladder scale according to groups and times.

Percentage changes in weight among the groups were analyzed, and it was found that the mean weight in group 4 (sham) was statistically higher than in groups GM1 24 hours and GM1 48 hours (p = 0.013 and p = 0.046, respectively). The results are shown in Fig. 4.

**Fig. 5:**
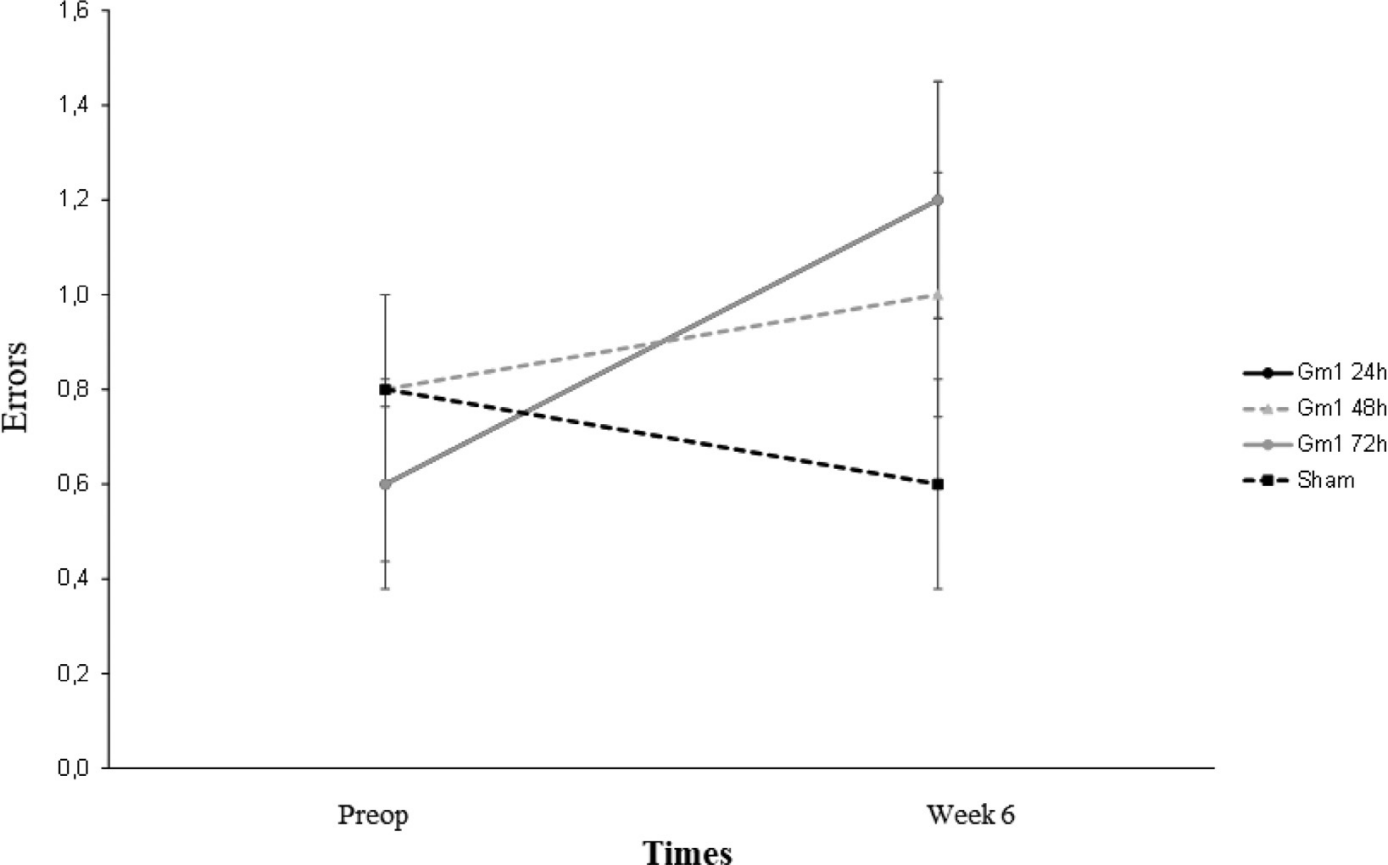
Mean values and respective standard errors of the errors on the horizontal ladder scale according to groups and times.

Fig. 5 shows that except the number of correct answers, all parameters evaluated in the horizontal ladder presented a mean statistically different throughout the evaluation times between the groups (p-interaction < 0.05), and the numbers of correct answers showed a mean statistically different throughout the evaluation, regardless of the group (p-moment = 0.002).

Table 2 shows that in all groups evaluated, there was a statistically significant decrease in the number of steps from preoperative to the sixth week (p < 0.05), and in the sixth week, the GM 1 48 hours group had a mean statistically significant of more steps than the other groups (p < 0.05).

**Table 2:** Results of the multiple comparisons of the steps among the groups and time.

						(95%) IC	
Group/Time	Comparison		Mean difference	Standard error	p	Inferior	Superior
Gm1 24h	Preop - Week 6		35.60	0.85	**<0.001**	33.09	38.11
Gm1 48h	Preop - Week 6		31.30	0.85	**<0.001**	28.79	33.81
Gm1 72h	Preop - Week 6		35.40	0.85	**<0.001**	32.89	37.91
Preop	Gm1 24h - Gm1 48h		0.00	0.81	>0.999	-2.38	2.38
	Gm1 24h - Gm1 72h		0.20	0.81	>0.999	-2.18	2.58
	Gm1 48h - Gm1 72h		0.20	0.81	>0.999	-2.18	2.58
Week 6	Gm1 24h - Gm1 48h		-4.30	0.81	**<0.001**	-6.68	-1.92
	Gm1 24h - Gm1 72h		0.00	0.81	>0.999	-2.38	2.38
	Gm1 48h - Gm1 72h		4.30	0.81	**<0.001**	1.92	6.68

Multiple comparisons of Bonferroni.

Tables 3 and 4 show that the slips and errors showed similar results among the groups throughout the evaluation times. In all groups, there was a mean statistically significant increase from preoperative to the sixth week (p < 0.05), and in the sixth week, the GM 1 48 hours group presented a mean statistically significant, fewer slips and errors than the other groups (p < 0.05).

**Table 3:** Results of the multiple comparisons of the slips among the groups and time.

						(95%) IC	
Group/Time	Comparison		Mean difference	Standard error	p	Inferior	Superior
Gm1 24h	Preop - Week 6		-36.20	0.82	**<0.001**	-38.61	-33.79
Gm1 48h	Preop - Week 6		-31.80	0.82	**<0.001**	-34.21	-29.39
Gm1 72h	Preop - Week 6		-36.10	0.82	**<0.001**	-38.51	-33.69
Preop	Gm1 24h - Gm1 48h		-0.10	0.83	>0.999	-2.55	2.35
	Gm1 24h - Gm1 72h		-0.10	0.83	>0.999	-2.55	2.35
	Gm1 48h - Gm1 72h		0.00	0.83	>0.999	-2.45	2.45
Week 6	Gm1 24h - Gm1 48h		4.30	0.83	**<0.001**	1.85	6.75
	Gm1 24h - Gm1 72h		0.00	0.83	>0.999	-2.45	2.45
	Gm1 48h - Gm1 72h		-4.30	0.83	**<0.001**	-6.75	-1.85

Multiple comparisons of Bonferroni.

**Table 4:** Results of the multiple comparisons of the errors among the groups and time.

						(95%) IC	
Group/Time	Comparison		Mean difference	Standard error	p	Inferior	Superior
Gm1 24h	Preop - Week 6		-36.20	0.98	**<0.001**	-39.06	-33.34
Gm1 48h	Preop - Week 6		-31.50	0.98	**<0.001**	-34.36	-28.64
Gm1 72h	Preop - Week 6		-36.00	0.98	**<0.001**	-38.86	-33.14
Preop	Gm1 24h - Gm1 48h		-0.20	0.93	>0.999	-2.94	2.54
	Gm1 24h - Gm1 72h		-0.20	0.93	>0.999	-2.94	2.54
	Gm1 48h - Gm1 72h		0.00	0.93	>0.999	-2.74	2.74
Week 6	Gm1 24h - Gm1 48h		4.50	0.93	**<0.001**	1.76	7.24
	Gm1 24h - Gm1 72h		0.00	0.93	>0.999	-2.74	2.74
	Gm1 48h - Gm1 72h		-4.50	0.93	**<0.001**	-7.24	-1.76

Multiple comparisons of Bonferroni.

## Discussion

The possibility of total spinal cord reconstruction still represents a challenge; however, it can obtain significant benefits through minimal repairs and regenerations. Although unable to fully recover gait, improvements such as the recovery of respiratory muscles, hand function, sphincter, and urinary control represent very important and extraordinary gains for these patients.^25^ The sequelae of spinal cord injury reflect significantly on the socioeconomic life of the families involved, and medicine continues looking for better viable therapeutic options to treat these patients. Even nowadays, medications are not well-established and proven to be effective. The strategies of immediate surgical decompression, supported and indicated by most physicians who treat these patients, as described in the last survey performed by AO Spine of 2022,^26^ encounter institutional difficulties due to financial or bureaucratic barriers. The use of corticosteroids is still fraught with great controversy and is known to be associated with adverse effects and complications.^27^, ^28^, ^29^, ^30^, ^31^ Therefore, other supportive measures include blood pressure maintenance, oxygen therapy,^32^ exercise, and continuous physiotherapy.^26^ The researchers continue looking for agents capable of decreasing, blocking, or stimulating the regeneration of the secondary injury area, referred to as the penumbra zone or apoptotic phase post-injury.^3^ It includes substances capable of delivering action that improve the inflammatory environment, controls neurotoxic agents, and release free radicals; for this, several types of research presenting different experimental animal models have been performed throughout history.^33^

The NYU-Impactor was established in the present institution as standard equipment for conducting research, using computerized equipment for the impact according to the parameters determined by the Multicenter Animal Spinal Cord Injury Study (MASCIS). As a result, it was possible to standardize and perform reproducible analyses to study the behavior and evolution of these injuries.

It can be observed throughout several studies of different anti-inflammatory agents, anti-oxidants, and with regenerative potentials that GM1 can stimulate the neural tissue neuroplasticity and regeneration pathways.

GM1 has been shown to be effective in improving neurological functions, with the ability to inhibit the evolution of the apoptotic phase after spinal cord injury, controlling the levels of caspase-3, a protein related to cell death mechanisms and GM1 being a natural lipophilic ganglioside of the central nervous system, the possibilities of adverse effects are more remote.^34^

The GM1 action may have a better effect when administered after acute spinal cord injury, as its actions are more related to the inhibition of the penumbra zone or secondary apoptotic injury.^35^ It inhibits the extracellular accumulation of glutamate and the excessive influx of sodium and calcium ionic ions mediated, initially, by the stimulation of NMDA receptors, with the subsequent evolution of the cell death area induced by the slow and gradual activation of AMPA receptors (glutamate kainate),^36^ leading to the maintenance of the influx of these ions, increasing the injury area in hours to days, with an estimated peak in three days.

Observing these mechanisms, the times at 24, 48, and 72 hours of GM1 applications after the induced spinal cord injury were established. It can be observed in clinical practice, evaluating the motor recovery of the animals, that the best result achieved was for the intrathecal applications of GM1 after 48 hours, demonstrating the best results after six weeks, especially in the horizontal ladder analyses. This result reinforces that its application after 24 hours, very close to the initial injury, can generate more inflammatory aggravation induced by the drug and the surgical approach for application than benefits, with no time of the apoptotic phase where GM1 would be more effective.

The time of 72 hours after the spinal cord injury coincides with the propagation of the apoptotic action where the penumbra zone would have already started. It shows the proximity of BBB scale results but shows differences in the horizontal ladder results, where it could be noticed greater numbers of steps and fewer slips in the application group in 48 hours.

The focus of the present study was on the clinical analysis of the animal's final evolution, with the already standardized BBB and horizontal ladder evaluation, since there are already studies, such as Ji et al. in 2015,^37^ demonstrating the effects of GM1 intrathecally, with extensive and well-illustrated immunohistochemical analyses after neurotoxicity induced by bupivacaine. Thus, through spinal cord injury, the present study evaluated the possibility of recovery and effective clinical improvement associated with the patterns of sensory and motor evaluations already known.^38^

Believing that GM1 would have the potential to mimic endogenous neurotrophic agents due to its ability to dimerize TRK receptors, activating neuroplasticity cascades,^39^ its role would also have a fundamental repercussion after neuronal injury since these neurotrophic factors are reduced.^35^

To perform such functions, GM1 depends on surpassing the blood-brain barrier, which is a limitation for lipophilic macromolecules, even under inflammatory conditions, when there are possibilities of greater transposition of this barrier.^13^^,^^40^ In the present study, the GM1 was applied to the central nervous system directly through established intrathecal injection in known and effective concentrations, defining the best timing for application and seeking the maximum possible result of this agent in its effective site of action.

The animal's weights were monitored and described in tables and graphs in the results section. After induced spinal cord injury, no animal had weight loss that placed it within the study's exclusion criteria.

One limitation of the present study was not having an imaging method to guide the procedures in animals in the laboratory, which imposed the need for new surgical approaches to each application, limiting the ability to expand the study. The authors believe that if there were an image-guided percutaneous way to maintain access, it would be easier to expand the sample and the results found.

With advances in experimental studies and publications of interesting and effective results in the recovery and treatment of these neurological injuries, the authors approach better therapeutic alternatives to minimize the suffering and limitations, which are often definitive, of these patients. Still, it is necessary to continue with more studies to expand the results and favor the use of these drugs and agents to be less harmful.

## Conclusion

The study demonstrated that the intrathecal application of GM1 after spinal cord contusion in Wistar rats is feasible. The application 48 hours after the injury presented the best functional results.

## Funding

No funding was received for this study.

## Data availability

The authors confirm that the data supporting the findings of this study are available within the article. Furthermore, the data sets used and/or analyzed during the current study are available from the corresponding author upon reasonable request.

## Ethics approval

This study was approved by Institutional Ethics Committee under the number 1402 and by the Ethics internal review boards of the Biosciences Institute under the number 1394/2019.

## CRediT authorship contribution statement

**Daniel de Moraes Ferreira Jorge:** Conceptualization, Investigation, Writing – original draft, Project administration. **Raphael Martus Marcon:** Validation, Writing – review & editing, Supervision, Project administration. **Alexandre Fogaça Cristante:** Project administration, Validation. **Tarcísio Eloy Pessoa Barros Filho:** Project administration, Validation. **Gustavo Bispo dos Santos:** Methodology, Investigation.

## Declaration of Competing Interest

The authors declare no conflicts of interest.
